# Improving the Understanding of Low Frequency Magnetic Field Exposure with Augmented Reality

**DOI:** 10.3390/ijerph191710564

**Published:** 2022-08-24

**Authors:** Florian Soyka, Julian Simons

**Affiliations:** 1 Department “Accident Prevention: Digitalisation—Technologies”, Institute for Occupational Safety and Health of the German Social Accident Insurance, D-53757 Sankt Augustin, Germany; 2 Faculty 4: Computer Science, Institute for Computational Visualistics, University of Koblenz and Landau, D-56070 Koblenz, Germany

**Keywords:** augmented reality, occupational safety and health, magnetic fields, visualization, exposure

## Abstract

Low frequency magnetic fields are often present in our everyday life due to the multitude of electronic devices. High magnetic fields can occur in the workplace from a wide variety of machines and systems, which must be measured and evaluated from the point of view of occupational safety. To facilitate the understanding of magnetic fields by supervisors and employees in the workplace, an augmented reality (AR) application was developed to visualize the measured flux densities and the resulting safety distances. The application was deployed on two smartphones, allowing for the simultaneous viewing of the same scene without the use of additional markers. Whether the application creates a better understanding of the exposure situation was evaluated with the help of an online survey. In this survey, participants received either a classic measurement report or a report enhanced by augmented images. The evaluation shows that it subjectively felt less difficult for participants with the augmented report to answer questions about the exposure situation. Furthermore, they also objectively performed better in answering the questions than did the group with the classic report. Therefore, this work shows that AR enhanced images can improve the understanding of an exposure situation, and it describes how such images and videos can be created.

## 1. Introduction

Low frequency magnetic fields are one of the many physical agents that can potentially be hazardous in a work environment [1]. In contrast to agents such as, for example noise, for which its presence and the potential need for protection is often rather obvious, magnetic fields are not detectable by humans without supplementary equipment. Therefore, employees are frequently unaware of the exposure and the potential hazard of this danger. Furthermore, once they become aware, there is still significant uncertainty about magnetic fields and their effects on humans. This uncertainty can result in being unnecessarily concerned or in being overly careless about exposure to this hazard. In our experience from evaluating workplaces for occupational safety and health (OSH) with respect to magnetic fields, we see that hazard communication can be difficult.

Images or sketches of the exposure situation might help employees to better understand the extent of the magnetic fields present and the safety distances which must be observed. However, creating such imagery can be a difficult task, since three-dimensional information about the exposure situation has to be visualized. Here, we present an augmented reality solution which embeds measurement and safety evaluation data directly into live imagery of the workplace being reviewed. Images or videos visualizing the exposure situation and the necessary safety distances can easily be created with this system. An evaluation study was performed investigating the influence of such imagery on the objective understanding of the exposure situation and on the subjective perception of the hazard communication. Evaluating information visualization is a challenging task in itself, and therefore, the study design took previous research into account [2]. This work was part of a master thesis and was done in collaboration with the University of Koblenz and Landau [3].

The aim of the system is to visualize data specifically for an OSH evaluation and not to visualize magnetic fields in general. Therefore, it is not necessary to visualize the field at any given point in time and space in its vectorial form (as e.g., in [4]). For an OSH evaluation, a conservative worst-case approach, focusing on maximum absolute values over time, is taken. Furthermore, a rather simple geometrical object (such as a box) is defined for providing safety distances, instead of defining a complicated volumetric object which exactly maps the magnetic field strength. In addition, care must be taken not to clutter the image, since visualizing too much information can also be distracting. For example, visualizing measurements taken along a line can show that the field strength decreases along with the distance from the source. This is true for all lines going away from the source, but showing too many measurements would not be helpful in that situation. This OSH-specific focus distinguishes our AR system from other solutions, e.g., the EM-Scanphone from Luxondes, which was designed with electromagnetic compatibility measurements in mind [5].

In general, AR technology becomes increasingly more feasible for practical use cases in industry settings and is estimated to continue growing to a ~USD 100 billion market by 2028 [6]. Its ability to combine spatial measurements with measurements of physical agents and to easily visualize this information offers great potential for OSH use cases, e.g., for acoustic measurements [7]. Which presentation format of AR content will be preferred by the end-users remains to be seen. Smartphones have the advantage of being widely available, whereas smart glasses, such as the Microsoft HoloLens, provide a high degree of immersion. Another possibility, which we explore here, is to create content (images or videos) with an AR system and subsequently present these familiar media in a classic report format. In this way, the end-user does not have to learn how to handle a new technology, but benefits from easy-to-understand imagery which could not have been created without the help of AR technology.

The aim of this work is to present the developed AR solution for visualizing magnetic fields in an OSH context and to show that media created with this system can improve the understanding regarding an exposure situation. Please note that the magnetic field measurement and assessment process can be very complex in itself. It is not the focus of this work and therefore, it is not described in detail, but is rather treated as a black box. The interested reader can find further information in [1].

## 2. Materials and Methods

This chapter is subdivided into two parts. The first part describes the setup of the AR measurement system, and the second part provides a description of the evaluation study.

### 2.1. The AR System for Visualizing Measurements and Safety Information

**Figure 1:**
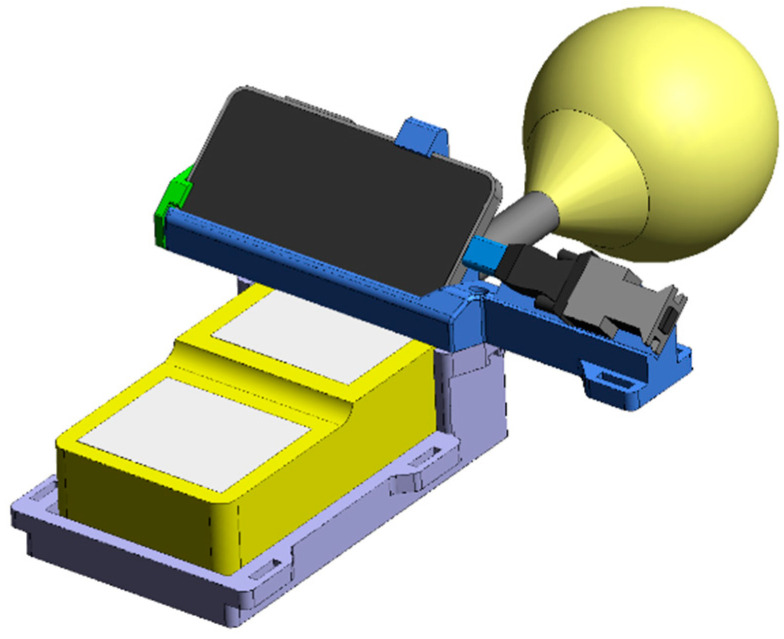
CAD drawing of the AR measurement system for magnetic fields. The yellow device with the spherical probe attached is the NARDA-EFA 300 measurement system, along with a standard size magnetic field probe. A custom designed 3D printed casing holds the EFA 300, with a SAMSUNG S20+ 5G smartphone running the AR application. The EFA 300 and the smartphone are connected via an optical cable and they communicate bidirectionally.

The main components of the AR system for visualizing magnetic field measurements and safety evaluation data are shown in Figure 1. The magnetic field measurements were performed with an EFA 300 system from NARDA, combined with a standard isotropic probe of a 100 cm^2^ area. The EFA 300 can measure magnetic fields for frequencies between 5 Hz and 32 kHz and with amplitudes between 100 nT and 32 mT. The typical uncertainty is specified as ±3%. In this study, peak values of the magnetic field were measured in broadband mode. A Samsung S20+ 5G smartphone was used for running the AR application. Bidirectional serial communication with an optical cable between the smartphone and the EFA 300 was established following the EFA 300 remote control protocol. A custom designed 3D printed casing combined the EFA 300 with the attached probe, the smartphone, and the necessary adaptors for the remote control connection in such a way that the probe was not visible in the field of view of the smartphone’s RGB and depth cameras. The whole system is called AURA—an abbreviation for augmented reality measurement system.

The software development was conducted with Unity [8]. Google’s freely available ARCore software development kit handles all augmented reality related calculations and provides the basis for the application [9]. ARCore provides an estimate of the pose (position and orientation) of the RGB camera in a world-coordinate system for each frame. Since the vector between the origin of the camera and the EFA 300 probe is fixed (due to the casing fixing the relative position of the components), it is easily possible to create AR objects at the position of the probe’s center.

In an initial “scanning” phase after starting the system, sensory data (visual features extracted from the RGB image, depth map from the time-of-flight camera, inertial measurement unit data) are gathered, while moving the AURA system around an object of interest until the ARCore system reports “good” localization accuracy. This is an ARCore internal accuracy estimate, and we chose to initially gather data and wait for “good” accuracy to be able to provide stable AR objects after the initial scanning phase. The achieved accuracy depends on many factors (visual features in the image, e.g., non-uniform surfaces, lighting conditions, stable surrounding, basically stable objects). In general, we achieved reasonably stable AR objects with our system (see Supplementary Materials Videos S1 and S2) allowing us to visualize measurement data within an average estimated accuracy of approximately five centimeters or less. For our given application, this was deemed sufficient, since the usual (non-AR assisted) measurement procedure involves similar measurement inaccuracies. Note that our aim was not to rigorously assess the accuracy of the system, but to generate imagery improving the understanding of the exposure situation. As AR technology evolves (better smartphones, sensors, and software solutions), it can be expected that the accuracy of the ARCore inside-out tracking will improve.

**Figure 2:**
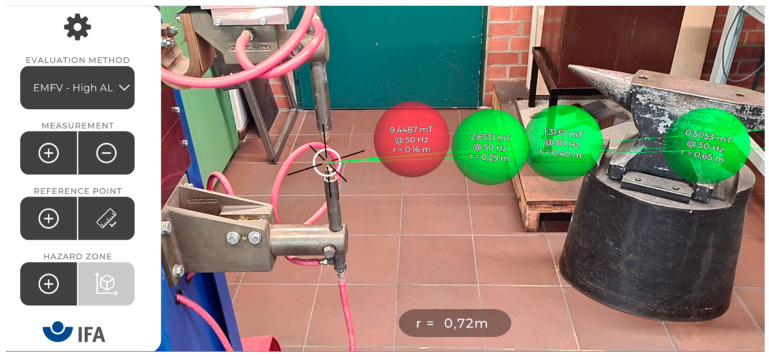
A typical measurement visualization regarding a resistance welding machine. The user interface on the left allows for the addition and removal of measurements (shown as colored spheres), or for the definition of a reference point (the white circle with the black axis). The coloring of the spheres indicates whether limit values are exceeded and depends on the selected evaluation method. The current distance to the reference point is shown at the bottom (e.g., r = 0.72 m).

Figure 2 shows the user interface and AR visualized measurements regarding a resistance welding machine (DALEX PL 63-4, 25 kA welding current, 100 ms welding time). The main features of the user interface are the adding and removing of measurements, setting a reference point, defining a hazard zone, and changing the safety evaluation method. AR visualizations need to be easy to understand and well embedded into the real world to be successful [10]. Therefore, measurements are visualized as slightly transparent spheres of the same size as the probe and at the position of the probe. The measured flux density and frequency are written inside the sphere, along with the distance to the reference point. A script continuously rotates the spheres so that the text always faces the camera. The measurement procedure is analogous to non-AR assisted measurements, in which a measurement point is defined with respect to a certain reference point. The actual measurement consists of the flux density, the dominant frequency, and the distance to the reference point.

The safety evaluation method can be chosen from a dropdown list containing different regulations, e.g., for workers with or without a pacemaker [11]. Depending on the active evaluation method, the spheres are color coded to visualize if a measurement is above a certain limit value, in which case the sphere would be red.

**Figure 3:**
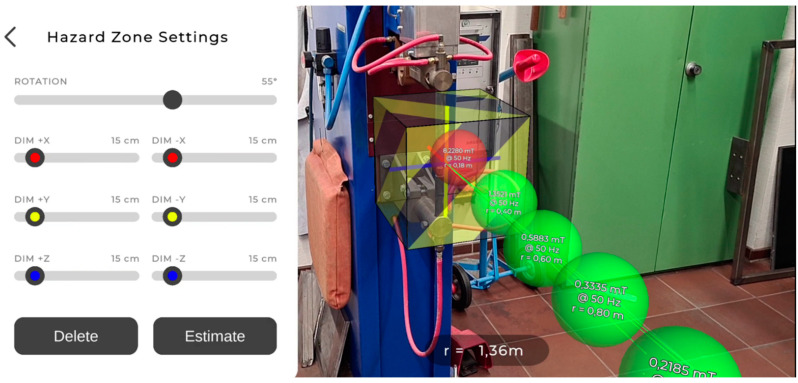
The hazard zone represents a volume within which the measured flux densities exceed a given limit value. In other words, it visualizes safety distances around the reference point. The dimensions can be estimated based on the measurements, and they can be manually adjusted.

Figure 3 shows the settings for defining a hazard zone, which represents a volume within which the measurements are above the limit values. In other words, the hazard zone represents safety distances. The zone is textured with half transparent black and yellow warning stripes, and the edges of the volume are highlighted in black. This rendering allows for good visibility of the hazard zone and provides a good understanding of how it is embedded in the environment. In general, AR objects can be (partially) occluded (due to the depth sensor data), which improves the 3D embedding of objects in the scene [12]. The hazard zone can be rotated around a vertical axis through its center, and its dimensions can be adjusted independently in 5 cm steps. This enables the occupational safety expert to quickly visualize the necessary safety distances in a workplace.

**Figure 4:**
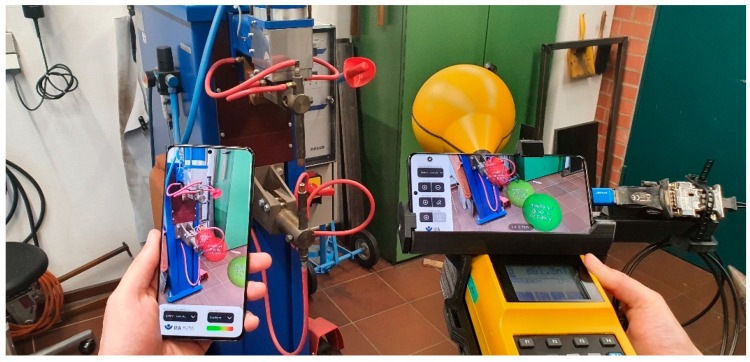
The Cloud Anchor functionality allows devices to share a common reference frame. By exchanging data about the objects in the scene, it becomes possible to view them independently from different perspectives with separate devices.

**Figure 5:**
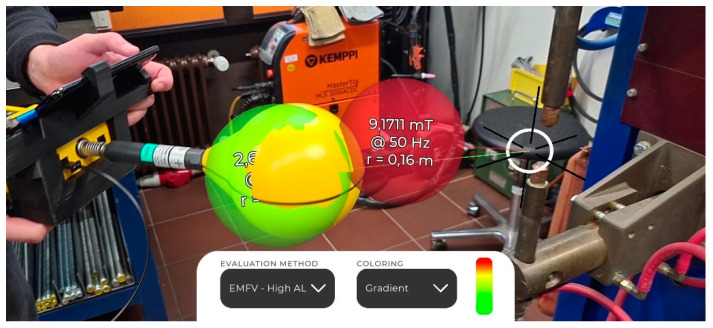
View of a measurement being taken as seen from the perspective of a second device. The virtual sphere representing the measurement coincides well with the measurement probe. The partial occlusion of the virtual sphere is possible due to the time-of-flight depth sensor in the smartphone.

In practice, many people are present during a magnetic field safety evaluation at a workplace. To allow them to explore the AR scene independently of the person with the AURA measurement system, the Cloud Anchors functionality of ARCore is used, which allows for viewing the same scene from a different perspective with another device (Figure 4). A Cloud Anchor can be understood as a collection of data which allows a device to recover the world coordinate system that was created during the initial scanning phase. The AURA application uploads the anchor, and the viewer application running on a second smartphone can subsequently get the Cloud Anchor from the internet. Furthermore, the AURA application uploads information about all AR objects into a Google Firebase Realtime Database [13]. Once the viewer application has resolved the world coordinate system from the Cloud Anchor, it can obtain the objects from the database and independently render the same scene from an arbitrary viewpoint. As an example, Figure 5 shows a measurement being performed from the perspective of the viewer application. This picture also gives an idea of the typically encountered accuracy of the system. The virtual sphere representing the measurement coincides well with the measurement probe.

The viewer application allows the user to change the evaluation method, which influences the coloring of the measurement spheres. Furthermore, the user can switch between a gradient and a binary (green/red) coloring scheme, indicating if a limit value is exceeded.

A video showcasing the AURA system and its capabilities can be found on the project web page [14] or in the Supplementary Materials (Video S1).

### 2.2. The Evaluation Study

The driving force behind developing the AURA system was to improve the understanding of magnetic field exposure situations by visualizing the measurements and the safety evaluation. From our practical experience in OSH, we know that people have difficulties understanding safety evaluation reports. The AURA system allows people to explore the exposure situation in AR. However, in practice, it is much more likely that people will read a report instead of being able to explore the AR scene with a smartphone at the workplace. Therefore, images (or potentially videos) augmented with the additional virtual information coming from the AURA system will probably be the main media which people will encounter.

To test if such augmented media could improve the understanding of a safety evaluation report, an online survey was conducted. A total of 2 groups of 15 participants each received a short report about the safety evaluation of magnetic fields from a resistance welding machine. Participants were recruited among colleagues and acquaintances. The study was anonymous, and participants acknowledged a data privacy statement. Participation was voluntary and was not compensated.

**Figure 6:**
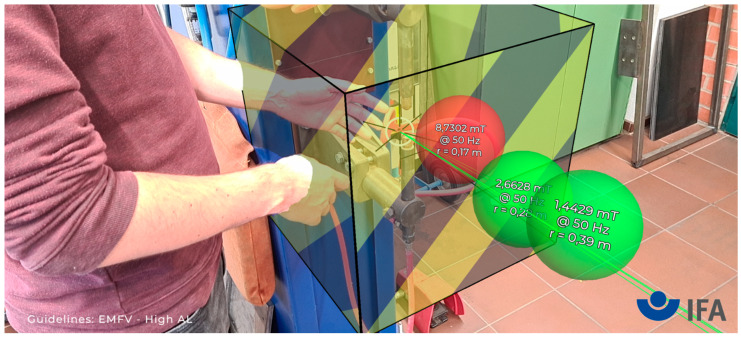
Twenty-eight of the thirty participants rated the AR visualization as either “helpful” or “very helpful” for improving the understanding of the exposure situation and the resulting safety distances.

The “Classic Report” group received a report which was similar to what is currently provided from OSH experts when evaluating a workplace (including result tables and images). The “Augmented Report” group received the same report, along with three additional images created with the AURA system, visualizing measurements and hazard zones (similar to Figure 6). After reading the report, both groups were required to answer questions about the exposure situation to evaluate their understanding of the report. Furthermore, they had to indicate from which chapter of the report they extracted the necessary information and how difficult it was for them to extract that information (on a 5-point Likert scale). Note that the AR enhanced images (only present for the “Augmented Report” group) were in a different chapter than the measurement results. Therefore, it is possible to check if the “Augmented Report” group extracted the information from the images rather than from the result tables.

A two sample *t*-test, with a 5% significance level, was performed in MATLAB on the results obtained from the 5-point Likert scale questions to test for differences between the groups.

In the end, both groups were shown an image (Figure 6) and a video (Supplementary Materials Video S2) created with the AURA system and asked if they thought that such media could be helpful for improving the understanding of the exposure situation and the safety distances (on a 5-point Likert scale).

## 3. Results

In the following, the “Classic Report” group will be abbreviated to “CR” group, and the “Augmented Report” group to “AR” group.

The mean age of the participants was 32.5 years. Seven of the thirty participants reported familiarity with magnetic fields in an OSH setting (four were from the “AR” group). Note that the survey was conducted in German.

**Figure 7:**
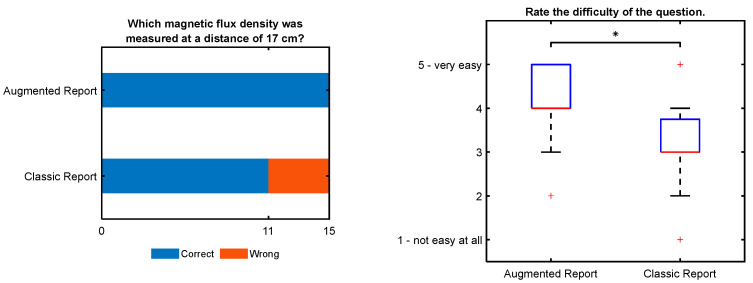
Participants in the “Classic Report” group made mistakes and perceived the question as significantly more difficult (indicated by the star). Six participants of the “AR” group reported that they used the AURA images to answer the question.

Figure 7 shows the results for the question, “Which magnetic flux density was measured at a distance of 17 cm?” It was a multiple-choice question for which one of seven possible answers could be selected. The correct answer could be found in a table in the report, or in the AURA images given only to the “AR” group.

All answers in the “AR” group were correct. Four of fifteen participants in the “CR” group answered incorrectly. Six participants of the “AR” group reported that they extracted the information from the AURA images.

Additionally, the participants were asked to rate the perceived difficulty of the question on a 5-point Likert scale. The results are shown as a box plot. There was a significant difference (*p* = 0.009) between the groups, showing that the “AR” group rated the task as less difficult.

**Figure 8:**
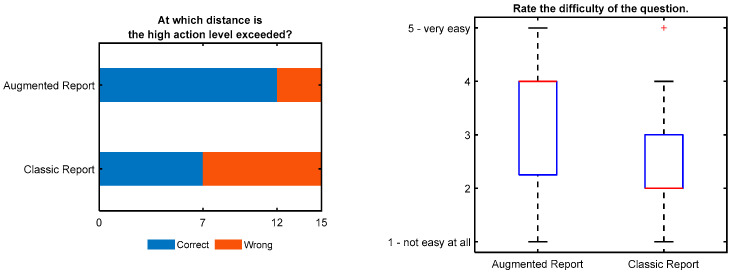
Results show that this question was difficult, and both groups made mistakes. This might be because the task involved comparing values across chapters. The “AR” group performed better, and there is a trend of perceiving the task as less difficult. Eight participants of the “AR” group reported that they used the AURA images to answer the question (six of them answered correctly).

Figure 8 shows the results for the question, “At which distance is the high action level exceeded?” It was a multiple-choice question for which one of seven possible answers could be selected. The correct answer could be found by comparing the measured values given in the results chapter with the limit value for the high action level given in the evaluation chapter. Furthermore, the “AR” group could infer the correct answer from a picture showing the measured values (color coded for the high action level) and the matching hazard zone.

Twelve answers in the “AR” group were correct, while only seven participants in the “CR” group answered correctly, which is less than half of the participants. Eight participants from the “AR” group reported that they extracted the information from the AURA images. However, two of these eight answered incorrectly.

Again, participants were asked to rate the perceived difficulty of the question on a 5-point Likert scale. There was no significant difference (*p* = 0.058) between the groups, but a strong trend towards the “AR” group rating the task as less difficult. The trend is supported by a median of 4 for the “AR” group and of 2 for the “CR” group.

**Figure 9:**
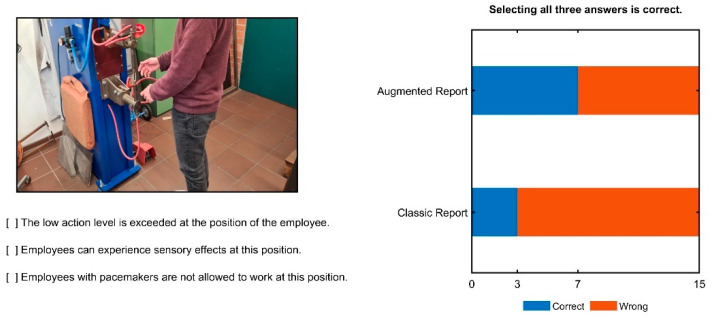
Three statements about the work position could be selected, and selecting all three correct statements equaled a correct response. This task required a good understanding of the exposure situation and the OSH context and therefore, was relatively difficult. In line with the other results, the “AR” group performed better.

Figure 9 shows the results for a task which required a good understanding of the exposure situation and the OSH context. The task was more general compared to the previous questions, which were rather specific in that they involved comparing numbers to tables.

Three statements about the picture were given, and the correct statements were to be selected. Selecting all three answers together equaled a correct response. At this stage, participants could not refer to the report anymore, but had to solve the task from memory with the knowledge they had acquired so far.

Only ten of all the thirty participants answered correctly. Seven were from the “AR” group and three from the “CR” group, which is in line with the other results. Interestingly, three of the seven people who indicated that they have experience with magnetic fields in an OSH context did not answer correctly.

In the last part of the survey, participants from both groups were shown an image (Figure 6) and a video (Supplementary Material Video S2) of measurements and a hazard zone (both created with the AURA system). They were asked to rate how helpful they think such media could be to improve the understanding of the exposure situation and the resulting safety distances. The image and the video were scored separately on a 5-point Likert scale from “not helpful at all” to “very helpful.” A total of 28 participants rated the image as either “helpful” or “very helpful” (27 for the video), which shows that most participants appreciated the AR visualization.

## 4. Discussion

The presented images and videos showcase that the AURA system can be used to easily create visualizations of measured flux densities and relevant safety information for workplace exposure scenarios. Almost all of the utilized software assets are freely available, and a high-end smartphone is sufficient for the intended task of achieving stability of the AR objects. Therefore, the AURA system represents a prototypical example, which can be improved and built upon without the need for proprietary knowledge.

The survey results show that AR enhanced images improve the understanding of the exposure situation, since AR participants made fewer mistakes when answering safety relevant questions about the exposure assessment report. Furthermore, there were significant differences in the perceived difficulty of the questions between groups, showing that participants provided with AR enhanced images felt that the questions were easier. These are very important and helpful results, since they show that it is worthwhile to create AR enhanced content. The results also indicate that the created content does not necessarily have to be presented in an interactive AR format to be helpful, which otherwise could be a technical challenge for some users. Instead, they show that classical media, such as images or videos, can benefit from AR enhancements.

It might seem surprising that the survey results revealed some wrong answers, even for “simple” tasks like extracting a number from a table. However, this is congruent with our experiences from OSH consultations. People often do not have a background in magnetic field health effects. This unfamiliarity with the topic, in combination with the safety evaluation aspect, tends to result in difficulties in understanding the provided exposure assessment reports. The results support these anecdotal experiences by quantitatively showing the difficulties participants had with answering some of the questions.

Being able to correctly answer questions such as, “At which distance is the high action level exceeded?” is very important. This knowledge is necessary to implement effective safety measures, like setting appropriate safety distances. The results show that AR enhanced images can help to reduce the number of mistakes; therefore, they can help to increase safety.

Another aspect we observe when showing AR enhanced imagery in an OSH setting is that people tend to take the hazard more seriously when they “see” it. Since magnetic fields cannot be seen, the potential threat is often neglected. However, this is not as easy to do when the hazard zone is visualized directly at the workplace.

The survey included an open feedback text field, and participants reported that they felt safer and more in control after seeing the AR enhanced imagery because they felt that they could better understand the necessary safety distances. Furthermore, they reported that seeing people directly in relation to the hazard zone (as in Figure 6) represents a new level in OSH imagery and is perceived as very helpful.

Future improvements to the AURA system could include playing a warning sound whenever the chosen limit value is exceeded by the currently measured value. However, movement of the probe within a magnetic field can induce voltages in the probe’s coils which in turn can lead to measurement artifacts. Therefore, measurements should only be taken when the probe is relatively steady in space. This would have to be considered when implementing a live measurement warning feature.

Electromagnetic compatibility of the smartphones when exposed to high magnetic fields is an important topic. High intensity fields could cause the devices to malfunction, or could damage them permanently. We could not find any data on frequency dependent magnetic field damage levels for smartphones. For the AURA system, it is important that the smartphones work at magnetic field levels which are in the range of the allowed limit values. Higher levels are not necessary since employees are not allowed to enter such areas anyway. Currently, we have used the AURA system at several workplaces, with different types of magnetic fields, and we have not yet encountered any electromagnetic compatibility issues (Table 1).

In general, it seems that AR enhanced imagery could also be helpful for agents other than magnetic fields. It could be helpful in the context of radiofrequency electromagnetic fields, e.g., visualizing hazard zones for wireless communication base stations. In that case, a concept very similar to the one presented here could be used by replacing the low frequency magnetic field measurement device with an appropriate high frequency measurement device.

Another application could be in the field of acoustics for noise measurements, e.g., as presented in [7]. In that case, a Microsoft HoloLens device could be used for presenting the AR imagery. It might be interesting to compare user acceptance for systems based on smart glasses with systems creating AR enhanced imagery, as in AURA.

Broadly speaking, AR visualization solutions can be helpful whenever a measured quantity has a room scale spatial dependency. Therefore, the concept lends itself very well to visualize all kinds of hazards in an OSH setting at a workplace.

## 5. Conclusions

In summary, we presented a novel system for visualizing magnetic flux density measurements for occupational safety in the workplace via augmented reality. Images and videos created with the system were used in an evaluation study to test whether such AR enhanced media can improve the understanding of the exposure situation. The results of an online survey showed that participants with access to such media made fewer mistakes answering safety relevant questions about the exposure. Furthermore, almost all participants perceived the AR enhanced content as very helpful. Therefore, we conclude that occupational safety and health workplace evaluations can benefit from AR enhanced media, and that such media can be created with reasonable effort given current technology.

## Figures and Tables

**Table 1:** The AURA system has been used at several different workplaces. So far, there have been no electromagnetic compatibility issues regarding the smartphones. This table shows the frequencies and the maximum measured flux densities of the encountered magnetic fields. The high action level limit value is given as a reference. The system must be able to measure at least this flux density to perform a meaningful assessment of the workplace.

Frequency (Hz)	Measured Flux Density (mT)	High Action Level (mT)
50	10	8.4
500	10	0.84
1500	6	0.28
8200	0.2	0.14

## Data Availability

The data presented in this study are available on request from the corresponding author.

## Supplementary Materials

The following supporting information can be downloaded at: https://www.mdpi.com/article/10.3390/ijerph191710564/s1, Video S1: Overview of the master thesis project—AR Visualization of Magnetic Fields; Video S2: Video shown at the end of the survey.
